# Supplementary material for: Smad4 and TGFβ1 dependent gene expression signatures in conditional intestinal adenoma, organoids and colorectal cancer
Source: Sci Rep. 2025 May 10;15:16330. doi: 10.1038/s41598-025-00908-4 (PMC12065906; doi:10.1038/s41598-025-00908-4)
Supplement: Supplementary file 10 — Supplementary Information 6. [file 41598_2025_908_MOESM10_ESM.pdf]

Smad4 and TGF-β1 dependent gene expression signatures in conditional intestinal adenoma, organoids and colorectal cancer.

Mirvat Surakhy[1], Julia Matheson[1], David Barnes[1,3], Emma J. Carter[1], Jennifer Hughes[1], Claudia Bühnemann[1], Sabina Sanegre[1], Hans Morreau[2], Paul Metz[1], Charlotte J. Imianowski[1] and A. Bassim Hassan[1,¶].

Supplementary Information Western Blots

Figure 1d
Western blot for anti-pSmad 1/5/8 and anti-pSmad3 and beta-actin

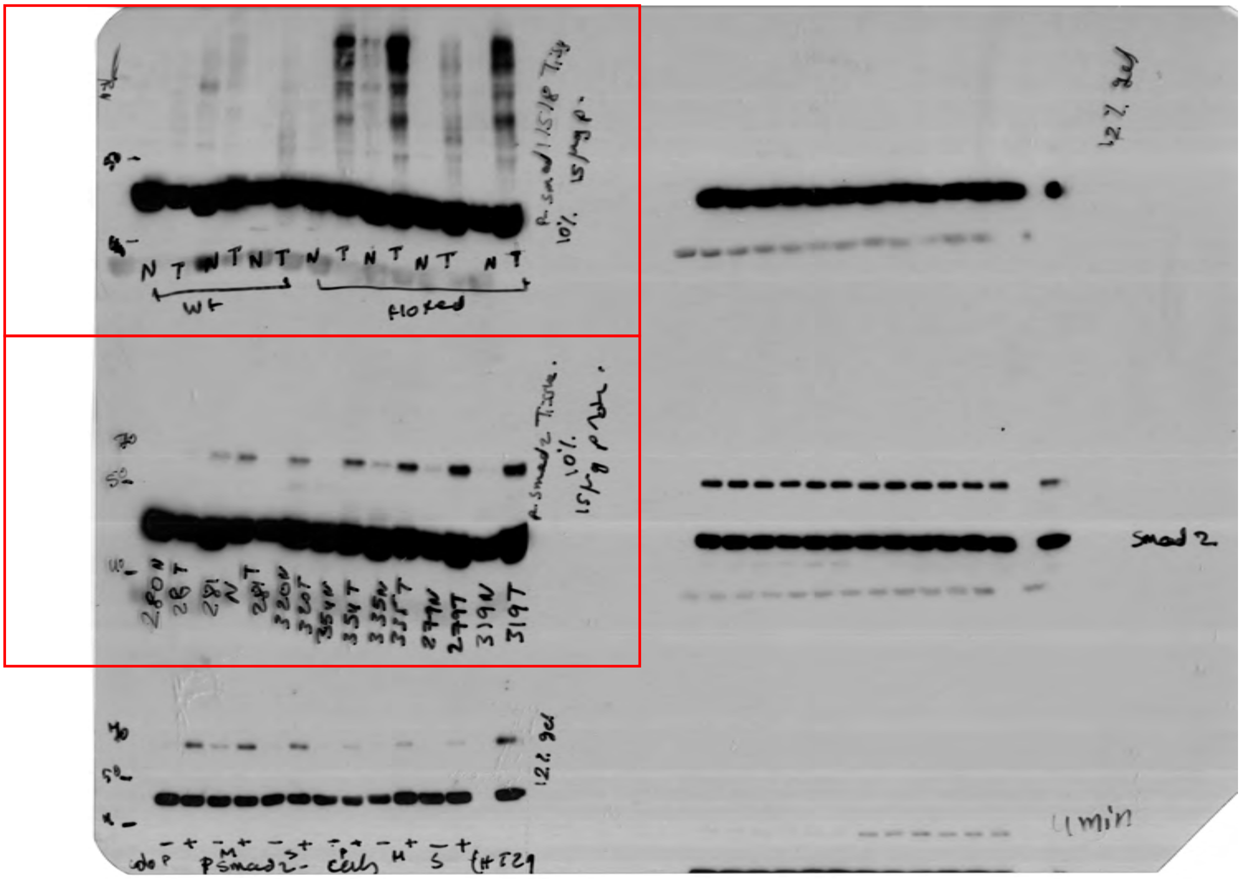

Western blot of adenoma (T) and normal tissue (N) from *Apc*$^{fl/fl}$*Smad4*$^{+/+}$ and *Apc*$^{fl/fl}$*Smad4*$^{f/fl}$ with anti-phospho-Smad2 (p-Smad2) and anti-phospho-Smad1/5/8 antibodies.

Figure 2c: Western blots for anti-pSmad2, anti-Smad2 and beta actin

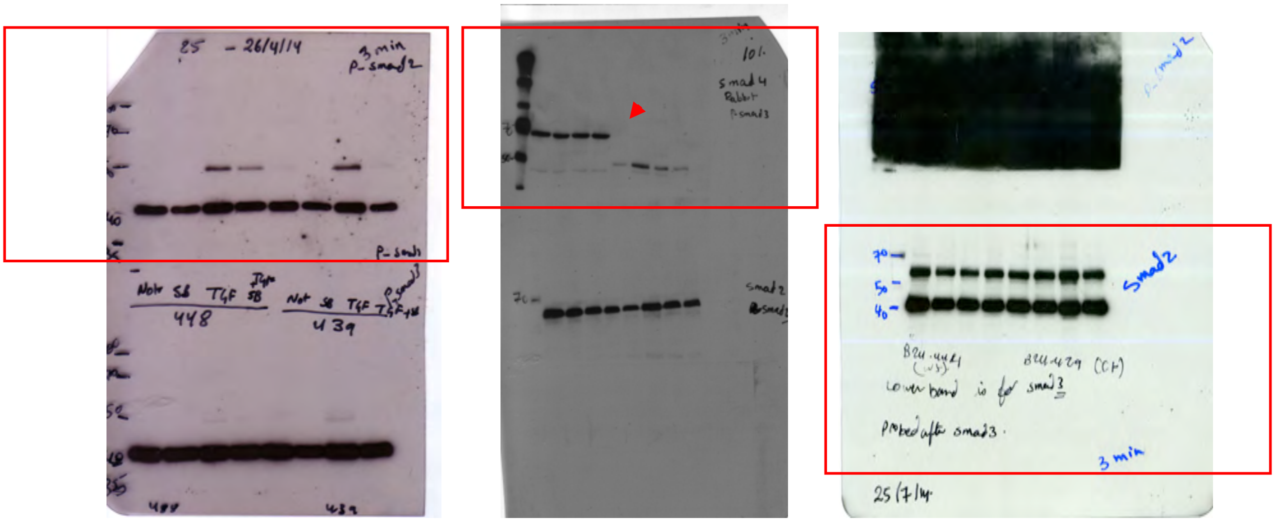

Western blots showing p-Smad2 (upper left), Smad4 (middle) and Smad2/ beta-actin (right). Lower left replicate for pSmad3 in Fig 2c. The order of the samples from left to right as follow: Lane 1-4 is Smad4 *Apc*$^{\Delta/\Delta}$*Smad4*$^{+/+}$ treated with vehicle, SB-505124, TGF-β1, and SB-505124, TGF-β1. And Lane 5-8 *Apc*$^{\Delta/\Delta}$*Smad4*$^{\Delta/\Delta}$ treated with vehicle, SB-505124, TGF-β1, and SB-505124, TGF-β1.

Figure 2c: Western blots for anti-pSmad3, anti-Smad3 and beta actin

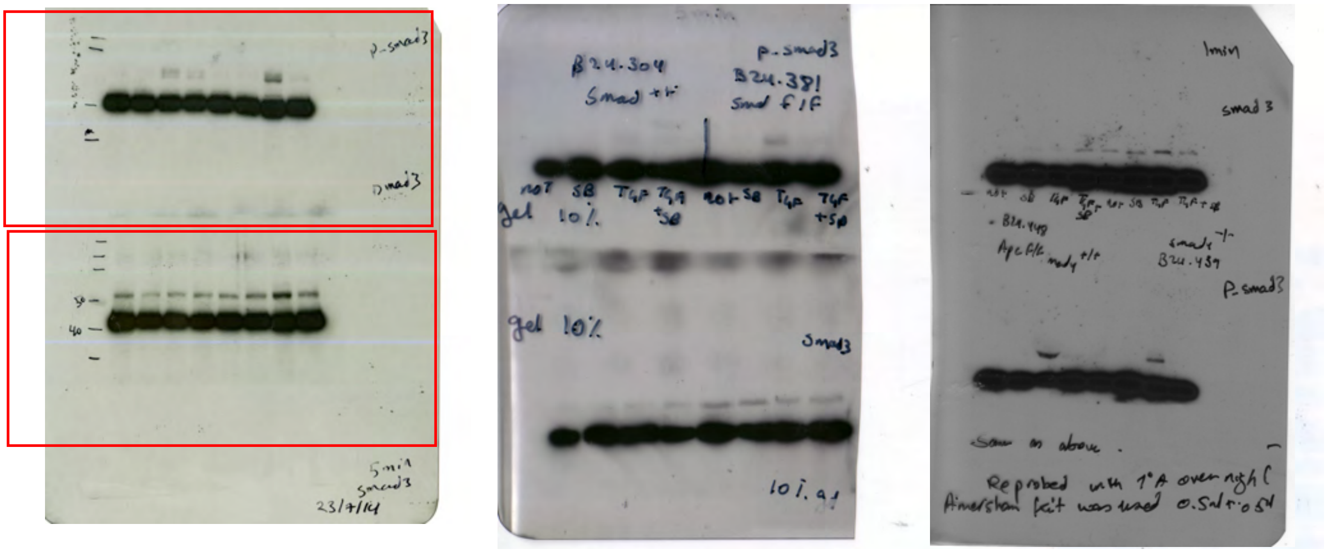

Western blots showing p-Smad3 (upper band) and Smad3 (Lower panel). The order of the samples from left to right as follow: Lane 1-4 is Smad4 *Apc*$^{\Delta/\Delta}$*Smad4*$^{+/+}$ treated with vehicle, SB-505124, TGF-β1, and SB-505124, TGF-β1. And Lane 5-8 *Apc*$^{\Delta/\Delta}$*Smad4*$^{\Delta/\Delta}$ treated with vehicle, SB-505124, TGF-β1, and SB-505124, TGF-β1. Middle and right panel replicates, with blot in Fig2c panel an additional replicate.

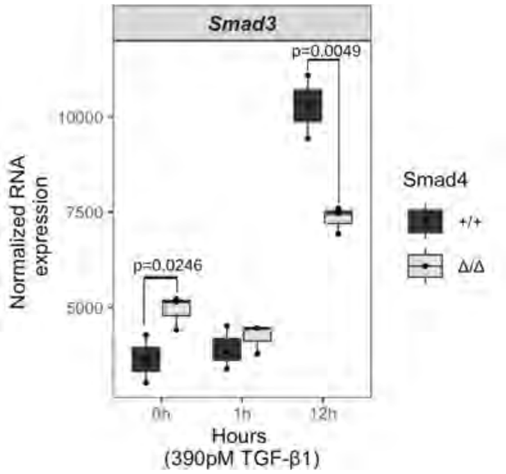

Effects of TGF-β1 exposure on Smad3 mRNA expression (as in Figure 3h).

Figure 2d
Western blot for anti-pSmad2 and beta-actin

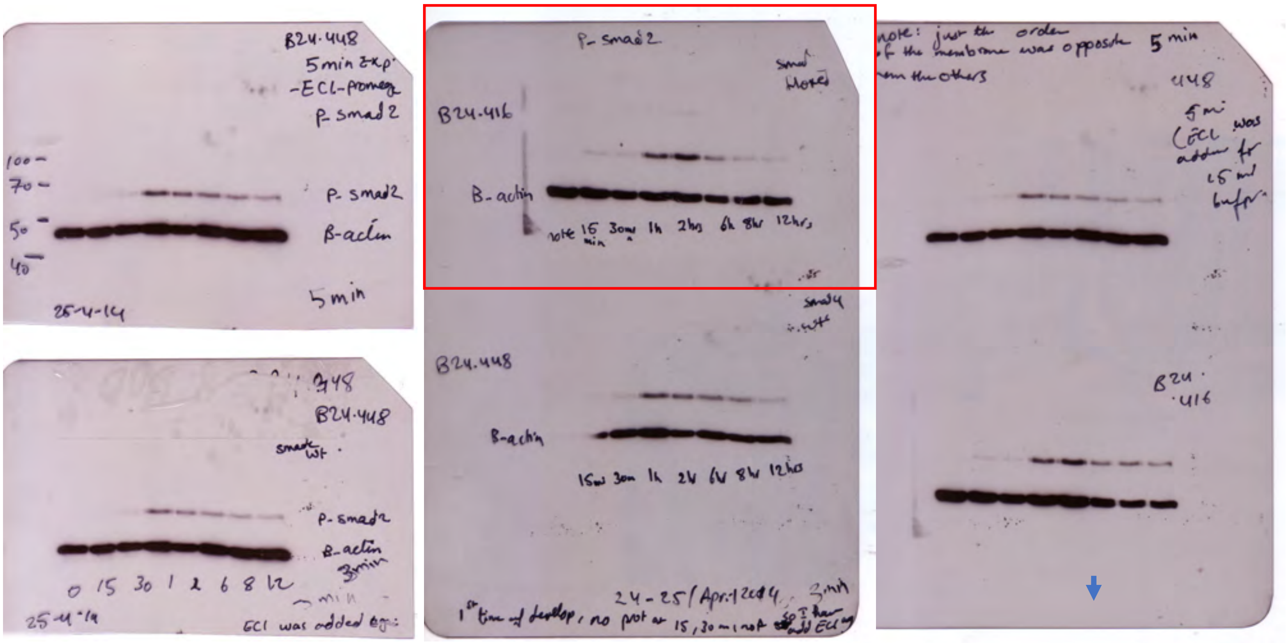

Associated western immunoblots of p-Smad2 at different time points (h, annotated) following TGF-β1 (390pM), densitometry relative to β-actin.

Figure 2g
Western blot for anti-Cleaved Caspase 3 (CC3)

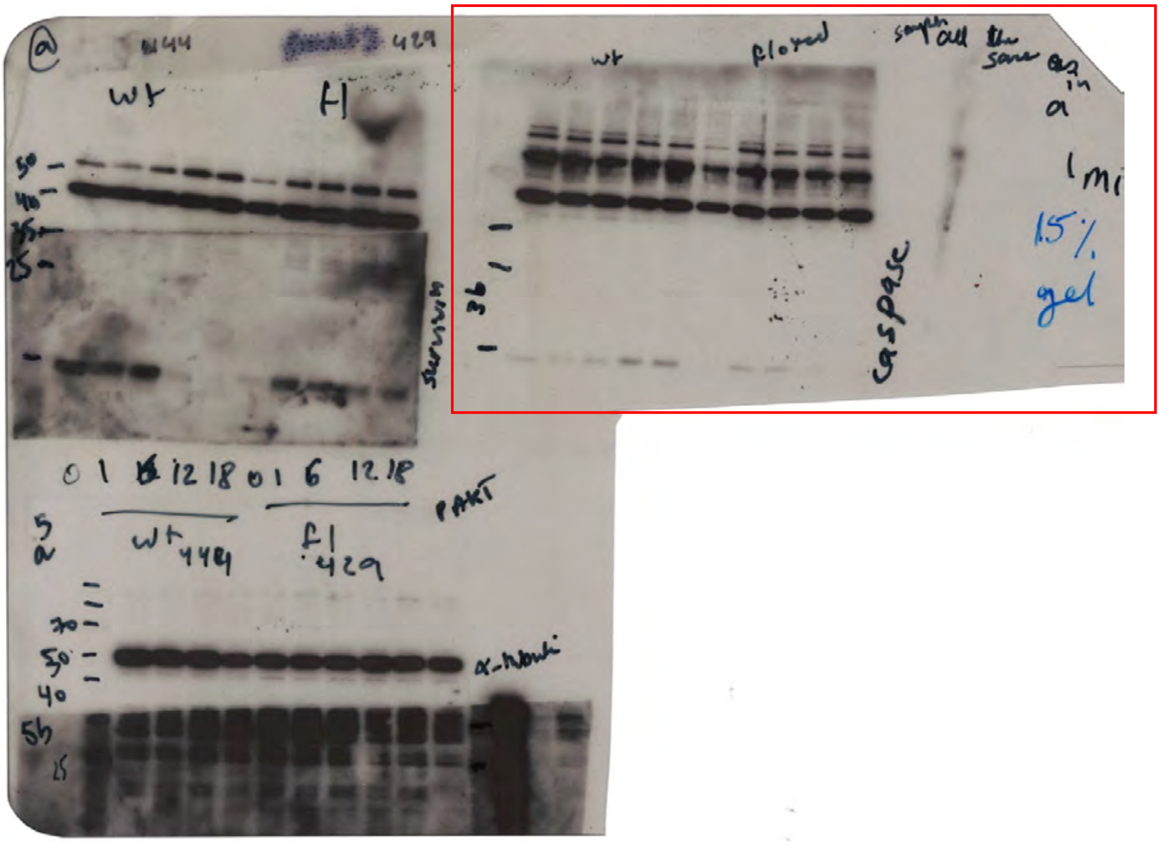

Associated western immunoblots of CC3 at different time points (h, annotated) following TGF-β1 (390pM), densitometry relative to β-actin.

Supplementary Figure 2c
Western blot for anti-Smad4 and anti-tubulin

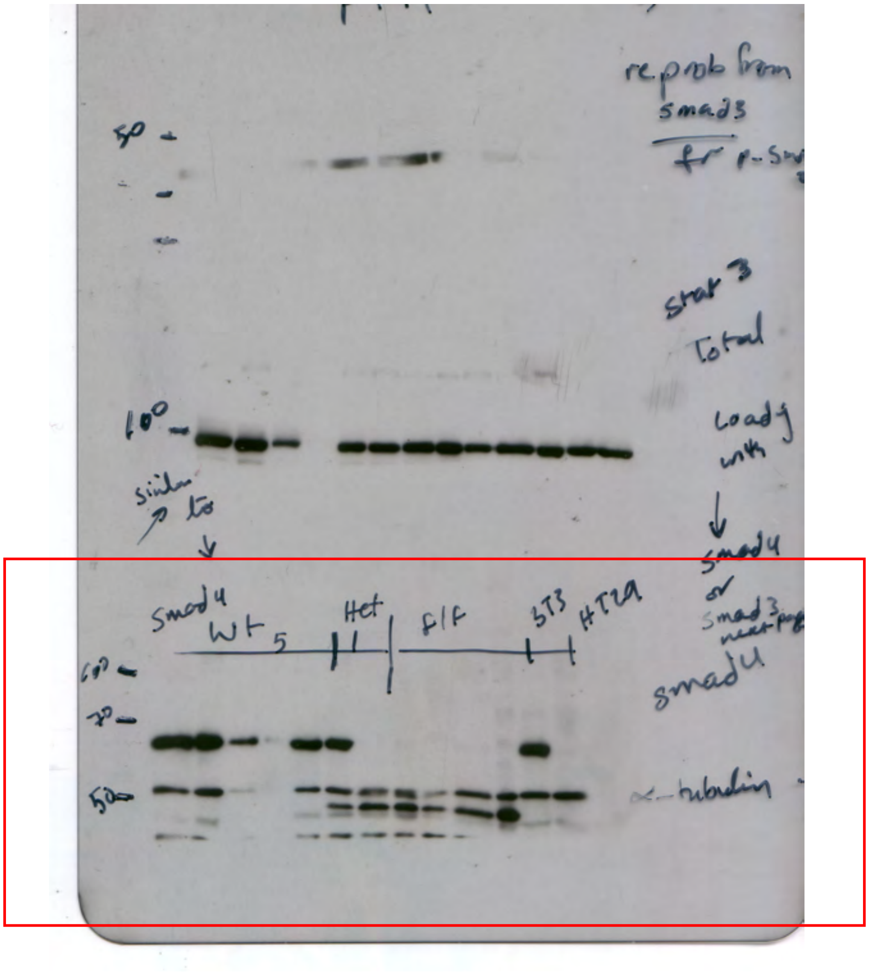

Western blots (left panel) showing Smad4 protein of 61kDa from *Apc*$^{Δ/Δ}$*Smad4*$^{+/+}$ and *Apc*$^{Δ/Δ}$*Smad4*$^{Δ/+}$ adenomas, and a truncated Smad4 protein of 43 kDa in *Apc*$^{Δ/Δ}$*Smad4*$^{Δ/+}$ and *Apc*$^{Δ/Δ}$*Smad4*$^{Δ/Δ}$. Controls: 3T3 fibroblast cells with intact *Smad4* (positive control). HT29 CRC cells have *Smad4* mutation (C931 C>T) in the MH2 domain (negative control).

Supplementary Figure 7b
Western blot for anti-Id1 and anti-beta-actin

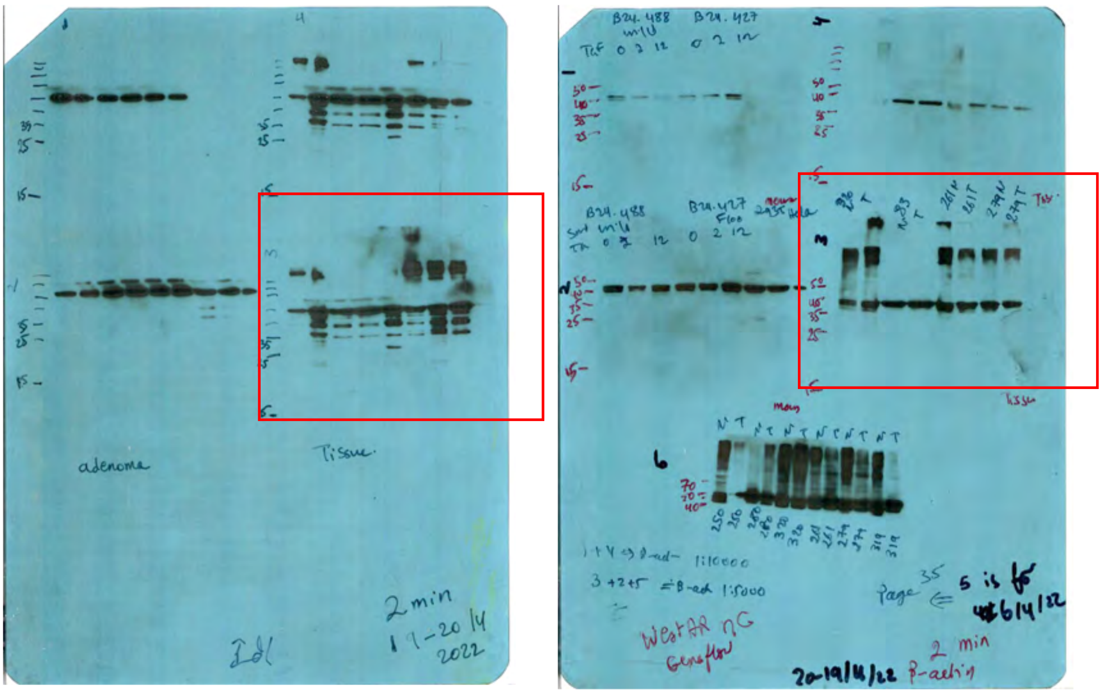

7b Western blot of Id1 (50kDa) from adenoma (T) and adjacent normal tissue (N) from *Apc*$^{fl/fl}$*Smad4*$^{+/+}$ and *Apc*$^{fl/fl}$*Smad4*$^{fl/fl}$. β-actin (43kDa) is a loading control.

Supplementary Figure 7c
Western blot for anti-Id1 and anti-beta-actin

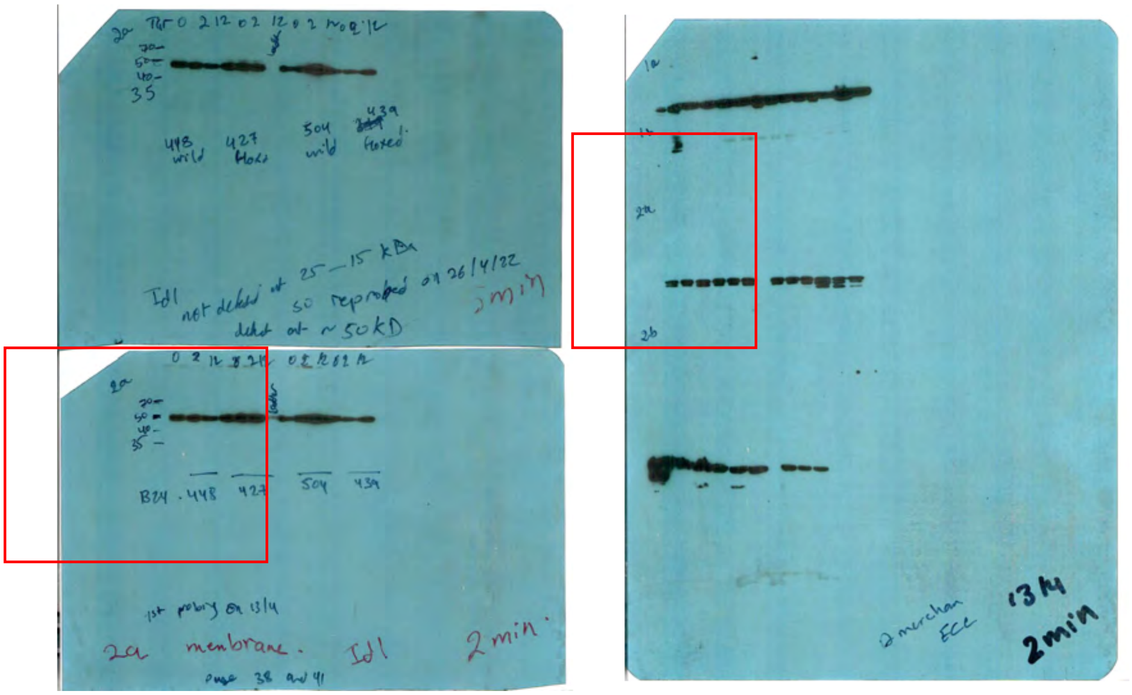

7c Western blot of Id1 from in *Smad4*$^{\Delta/\Delta}$ and *Smad4*$^{+/+}$ adenoma organoids at t=0, t=2 and t=12hr of 390pM TGF-β1 treatment.

Supplementary Figure 8
Western blots for anti-fibronectin, anti-N-cadherin, anti-Smad4, anti-c-Myc and anti-beta-actin

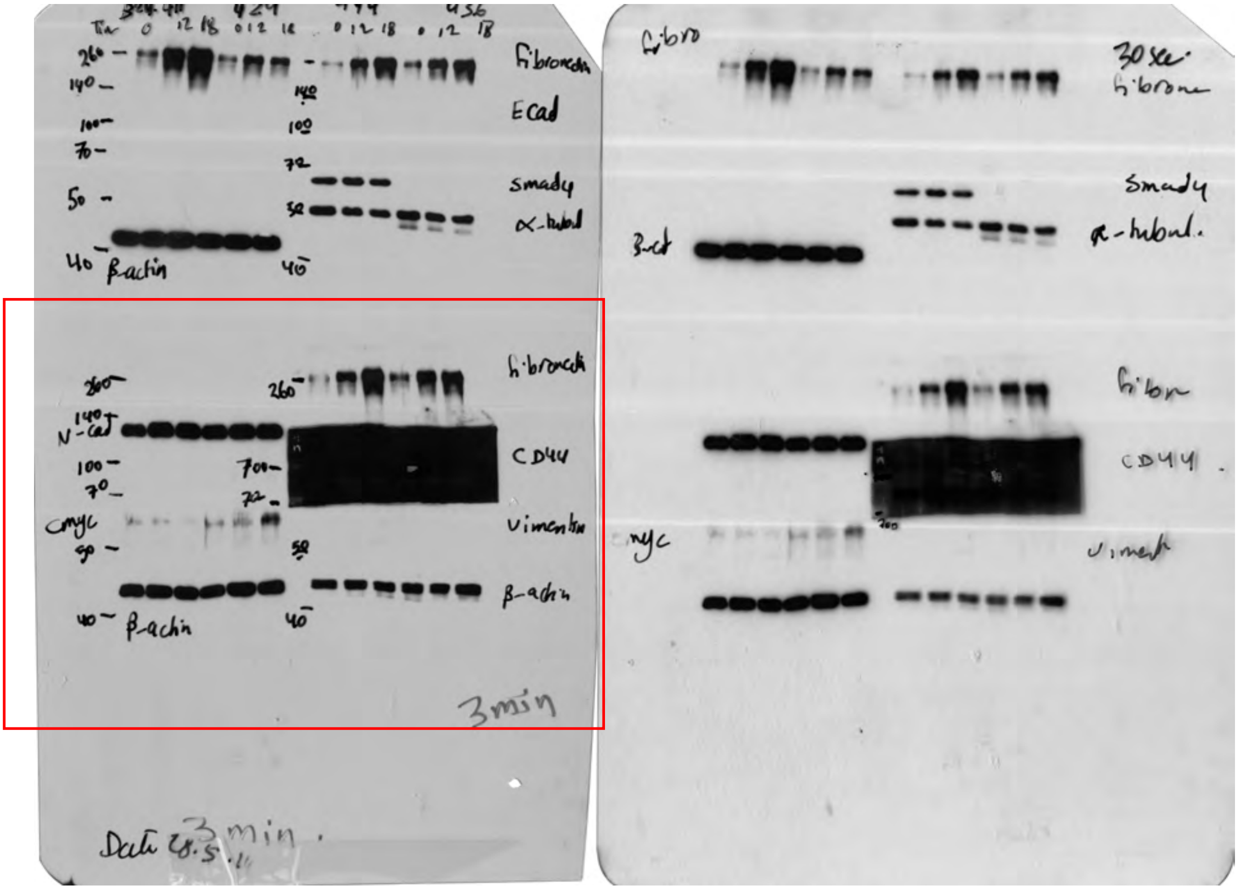

Western blot used for Suppl Figure 8 for fibronectin, N-cadherin with β-actin, also included C-myc and Smad4.

Supplementary Figure 9
Western blots for anti-survivin and anti-beta-actin

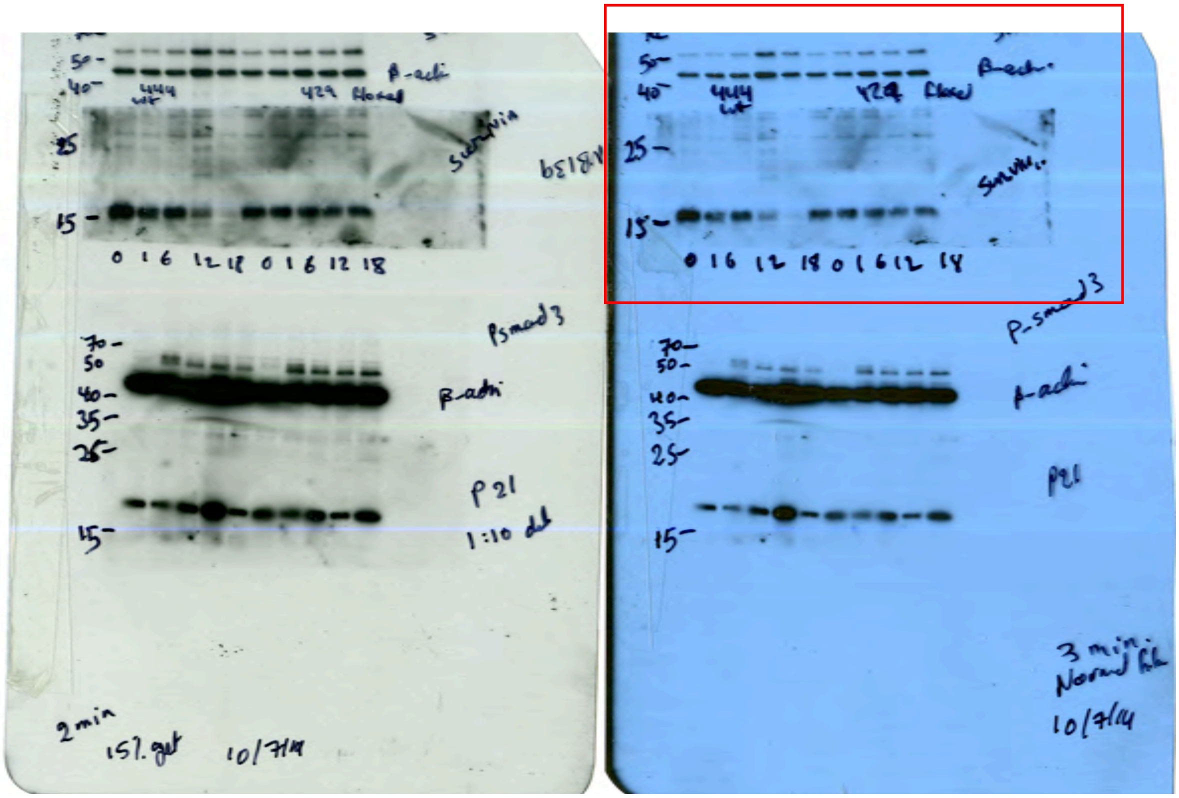

Western immunoblots showing the downregulation of Survivin within 12h TGF-β1 treatment predominantly in Smad4$^{+/+}$ adenomas.

Supplementary Figure 10
Western blots for anti-beta catenin, E-cadherin and C-Myc with anti-beta-actin

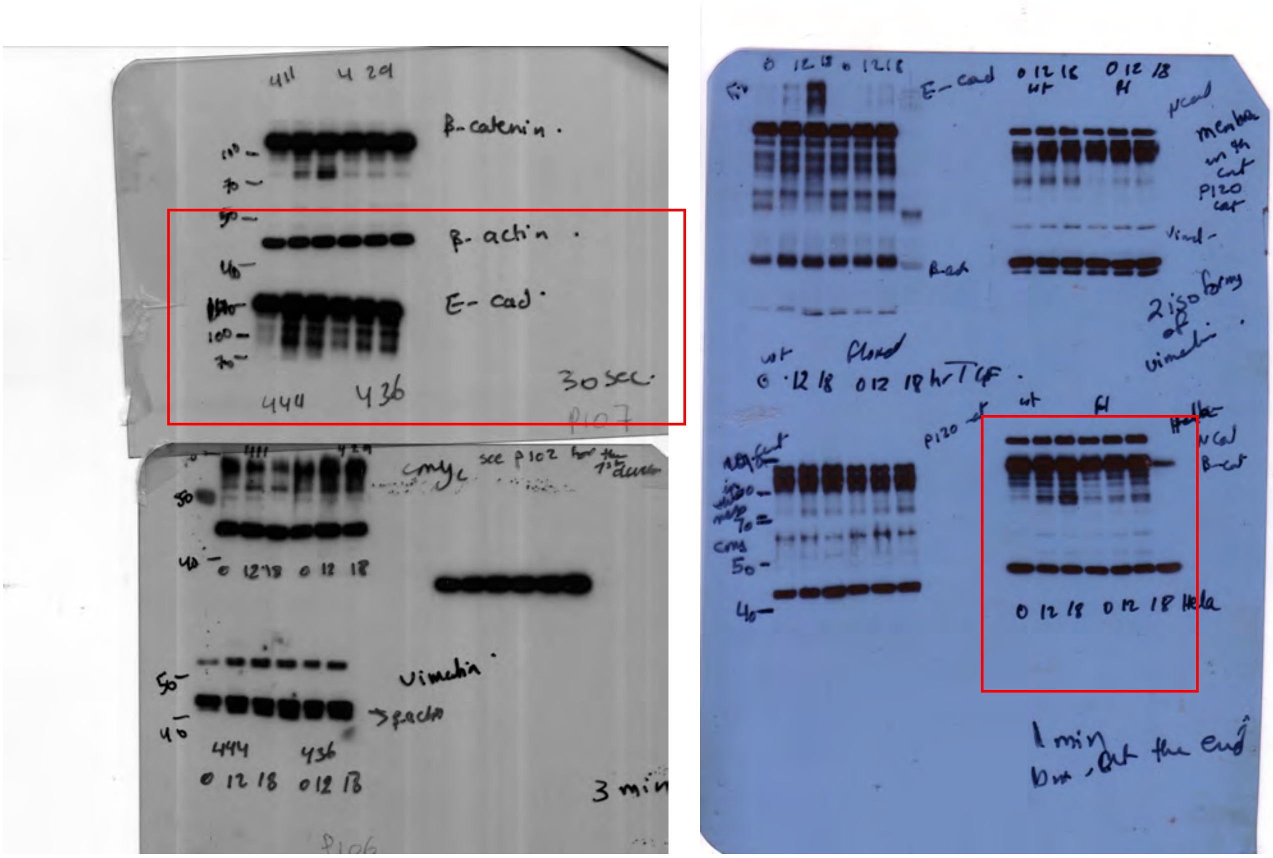

Representative western blots showing for β-catenin, E-cadherin and c-Myc at different time points following TGF-β1 exposure. β-catenin at 92 kDa (arrow) with multiple bands suggesting degradation. E-cadherin at 130 KDa with multiple bands suggesting degradation. c-Myc at 67 KDa.
