# Supplementary material for: Smad4 and TGFβ1 dependent gene expression signatures in conditional intestinal adenoma, organoids and colorectal cancer
Source: Sci Rep. 2025 May 10;15:16330. doi: 10.1038/s41598-025-00908-4 (PMC12065906; doi:10.1038/s41598-025-00908-4)
Supplement: Supplementary file 13 — Supplementary Information 9. [file 41598_2025_908_MOESM13_ESM.pdf]

Smad4 **and TGFβ1 dependent gene expression signatures in conditional intestinal adenoma, organoids and colorectal cancer**

Mirvat Surakhy[1], Julia Matheson[1], David Barnes[1,3], Emma J. Carter[1], Jennifer Hughes[1], Claudia Bühnemann[1], Sabina Sanegre[1], Hans Morreau[2], Paul Metz[1], Charlotte J. Imianowski[1] and A. Bassim Hassan[1,¶].

[1]Oxford Molecular Pathology Institute, Sir William Dunn School of Pathology, University of Oxford, South Parks Road, Oxford, OX1 3RE, United Kingdom.
[2]Department of Pathology, Leiden University Medical Centre, Leiden, The Netherlands.
[3]Institute of Cancer and Genomic Sciences, University of Birmingham, Birmingham B15 2TT

Supplementary Tables

Supplementary Table 1

Differentially Expressed Genes (DEG) in *Apc*$^{\Delta/\Delta}$*Smad4*$^{+/+}$ and *Apc*$^{\Delta/\Delta}$*Smad4*$^{\Delta/\Delta}$ adenomas at different time of TGF-β treatment. Supplementary sheets present DEGs identified in pairwise comparisons between *Smad4*$^{\Delta/\Delta}$ and *Smad4*$^{+/+}$ organoids at different time points (0h, 1h, and 12h). Log_2 fold change (Log_2FC) and adjusted p-values (Padj) were calculated to determine significant gene expression differences using Deseq2. a-c) DEGs in *Smad4*$^{\Delta/\Delta}$ cells compared to *Smad4*$^{+/+}$ cells at corresponding time points. d-f) DEGs in *Smad4*$^{+/+}$ cells across different time points. g-h) DEGs in *Smad4*$^{\Delta/\Delta}$ cells across different time points. i) Summary table providing Log_2FC and Padj for all eight pairwise comparisons.

Table 1a: DEG in Smad4Δ/Δ 0h compared to Smad4+/+ 0h.
Table 1b: DEG in Smad4Δ/Δ 1h compared to Smad4+/+ 1h.
Table 1c: DEG in Smad4Δ/Δ 12h compared to Smad4+/+ 12h.
Table 1d: DEG in Smad4+/+ 1h compared to Smad4+/+ 0h.
Table 1e: DEG in Smad4+/+ 12h compared to Smad4+/+ 0h.
Table 1f: DEG in Smad4+/+ 12h compared to Smad4+/+ 1h.
Table 1g: DEG in Smad4Δ/Δ 12h compared to Smad4Δ/Δ 0h.
Table 1h: DEG in Smad4Δ/Δ 12h compared to Smad4Δ/Δ 1h.
Table 1i: Summary of Log_2FC and adjusted p-value for the eight pairwise comparisons.

Supplementary Table 2

Pathway Enrichment Analysis Using MSigDB Hallmark Gene Sets and tmod R package: 2a) Summary of pathway enrichment analysis results using the CERNO test in tmod package. The table shows the combined rank-based enrichment score from the CERNO test (cerno), shows area under the curve (AUC), combined enrichment score (cES), number of the genes in each module (N1), p-value, and adjusted p-value (adj.P.Val). 2b) List of genes contributing to the enrichment of pathways identified in tmod analysis. Pathways and genes with an adjusted p-value < 0.05 were considered significantly enriched.

Table 2a: Pathway enrichment analysis using MSigDB Hallmark gene sets with tmod.
Table 2b: Genes enriched in tmod analysis from MSigDB Hallmark gene sets.

Supplementary Table 3

TGF-β Response and Smad4-Dependent Genes. This table summarises genes associated with the TGF-β signalling pathway, categorised by early and late response, as well as Smad4-dependent and independent regulation in *Smad4*$^{+/+}$ adenomas. Log_2FC and Padj are provided for differentially expressed genes. 3a–3c) TGF-β early and late response genes that are upregulated or downregulated in *Smad4*$^{+/+}$ adenomas. d–e): Genes classified as Smad4-independent or Smad4-dependent, including their name, description, and regulatory direction (see Method). f) Genes regulated at baseline (0h), irrespective of TGF-β signalling, with their respective descriptions and directions of regulation.

Table 3a: TGF-β Early response genes upregulated in Smad4+/+ adenomas (Log_2FC and adjusted p-value).
Table 3b: TGF-β late response genes upregulated in Smad4+/+ adenomas (Log_2FC and adjusted p-value).
Table 3c: TGF-β late response genes downregulated in Smad4+/+ adenomas (Log_2FC and adjusted p-value).
Table 3d: TGF-β Smad4 independent Genes (gene name, description, and direction).
Table 3e: TGF-β Smad4 dependent Genes (gene name, description, and direction)
Table 3f: Genes regulated at the baseline (gene name, description, and direction).

Supplementary Table 4
Marker Genes Identified from Single-Cell RNA Sequencing (scRNA-seq) Analysis: This table presents marker genes identified from scRNA-seq clustering analysis at resolutions 1.2 for all clusters (a) and resolution 2.2 for cluster 10 and 22 (b). Each table shows the Log_2 fold change of the gene expression between the given cluster and the rest of the dataset (avg_log_2FC), proportion of cells expressing the gene within the cluster (pct.1), proportion of cells expressing the gene in all other clusters (pct.2), adjusted p-value for multiple testing correction, cluster number (cluster) and gene name.

Table 4a: Marker gene for all clusters from the single cell RNA-seq at 1.2 resolution.
Table 4b: Marker gene for clusters 10 and 22 from the single cell RNA-seq at 2.2 resolution.

Supplementary Table 5
Statistical Analysis of Gene Expression in TCGA CRC Cohorts Based on *SMAD4* Mutation Status. This dataset presents statistical analyses of gene expression, *Smad4* signature in mouse adenoma, across COAD and READ TCGA cohorts. a) Mann-Whitney p-values for genes evaluated in TCGA cohorts, adjusted for false discovery rate (FDR) (see Figure 5b). b) patient numbers categorised by pathway and Smad4 mutation status (see Figure 5a). The fold difference in *ID1* RSEM median expression between different groups was analysed using Dunn's multiple comparisons test with Holm p-adjustment. Kruskal-Wallis p-values and FDR adjustment provided for the analysis of all 78 genes (Sheet d). d) Kruskal-Wallis p-values for genes in the context of *SMAD4* mutation, with FDR adjustment. e) Multiple Comparisons (Dunn Test) for genes with significant Kruskal-Wallis adjusted p-value (from sheet d). f) Significant DEGs ($Log_2FC > |1.5|$ and adjusted p-value < 0.05) in $Smad4^{\Delta/\Delta}$ mouse adenomas compared to *Smad4*$^{+/+}$ at 0h, with human-HGNC counterparts in TCGA cohorts. g) Comparison of *ID1*, *SPP1*, and *PAK3* expression between different groups (see Figure 6a). h) TCGA cohorts with the mutation status used in this study.

Table 5a: Patients number in the study groups.
Table 5b: Fold difference in the RSEM median expression of *ID1* between different groups.
Table 5c: Fold difference in the RSEM median expression of *ID1* between different groups with no BMP pathway mutation.
Table 5d: Kruskal-Wallis p-values for genes evaluated in the TCGA cohorts in the context of SMAD4 mutation.
Table 5e: Multiple comparisons for genes with significant Kruskal-Wallis comparison (Dunn test).
Table 5f: Significant genes in adenoma at 0h with absolute $Log_2FC > 2$ and adjusted p-value <0.05 and their human orthologs.
Table 5g: Comparison of *ID1, SPP1, PAK3* expression between different groups in TCGA cohorts.
Table 5h: TCGA cohorts with the mutation status used in the study.
